# Genome-Wide Survey and Expression Profiling of CCCH-Zinc Finger Family Reveals a Functional Module in Macrophage Activation

**DOI:** 10.1371/journal.pone.0002880

**Published:** 2008-08-06

**Authors:** Jian Liang, Wenjun Song, Gail Tromp, Pappachan E. Kolattukudy, Mingui Fu

**Affiliations:** Biomolecular Science Center, College of Medicine, University of Central Florida, Orlando, Florida, United States of America; Center for Genomic Regulation, Spain

## Abstract

Previously, we have identified a novel CCCH zinc finger protein family as negative regulators of macrophage activation. To gain an overall insight into the entire CCCH zinc finger gene family and to evaluate their potential role in macrophage activation, here we performed a genome-wide survey of CCCH zinc finger genes in mouse and human. Totally 58 CCCH zinc finger genes in mouse and 55 in human were identified and most of them have not been reported previously. Phylogenetic analysis revealed that the mouse CCCH family was divided into 6 groups. Meanwhile, we employed quantitative real-time PCR to profile their tissue expression patterns in adult mice. Clustering analysis showed that most of CCCH genes were broadly expressed in all of tissues examined with various levels. Interestingly, several CCCH genes Mbnl3, Zfp36l2, Zfp36, Zc3h12a, Zc3h12d, Zc3h7a and Leng9 were enriched in macrophage-related organs such as thymus, spleen, lung, intestine and adipose. Consistently, a comprehensive assessment of changes in expression of the 58 members of the mouse CCCH family during macrophage activation also revealed that these CCCH zinc finger genes were associated with the activation of bone marrow-derived macrophages by lipopolysaccharide. Taken together, this study not only identified a functional module of CCCH zinc finger genes in the regulation of macrophage activation but also provided the framework for future studies to dissect the function of this emerging gene family.

## Introduction

Macrophage activation is an essential cellular process underlying innate immunity, enabling the body to combat bacteria and other pathogens. In addition to host defense, activated macrophages play a central role in atherogenesis, autoimmunity, and a variety of inflammatory diseases [1], [2]. Much attention has focused on the pro-inflammatory signaling in activated macrophages but little is known about the mechanisms that negatively control inflammation. Understanding the molecular mechanisms involved in the inflammatory processes in macrophages is essential for development of novel drug therapies against inflammatory diseases including atherosclerosis.

Previously, we have identified a novel CCCH zinc finger protein, which was significantly induced by MCP-1 in human monocytes and thus designated as MCP-induced protein (MCPIP), as negative regulator of macrophage activation [3], [4]. The CCCH-zinc finger was originally identified in tristetraprolin (TTP, also known as Zfp36) family, which contains four members Zfp36, Zfp36l1, Zfp36l2 and Zfp36l3. This protein family contains two tandem CCCH-zinc fingers and binds to AU-rich elements (ARE) in mRNA, leading to the removal of the poly(A) tail from that mRNA and increased rates of mRNA turnover [5]–[7]. TTP-deficient mice developed a systemic inflammatory syndrome with severe polyarticular arthritis and autoimmunity, as well as medullary and extramedullary myeloid hyperplasia due to excess circulating TNFα, resulting from the increased stability of the TNFα mRNA and subsequent higher rates of secretion of the cytokine [8]. Zfp36l2, like its better-known relative Zfp36, is also a mRNA-binding and destabilizing protein and functions in the physiological control of female fertility at the level of early embryonic development [9]. Zinc-finger antiviral protein (ZAP, also known as Zc3h2), which is also a CCCH-type zinc finger protein, can directly bind to specific viral RNA sequences through its CCCH zinc finger motifs and cause profound and specific loss of viral mRNA [10], [11]. Rc3h1 (also known as Roquin) is a conserved protein containing both a ring-finger and a CCCH-type zinc finger. Recent studies revealed that roquin repressed autoimmunity by limiting inducible T-cell co-stimulator mRNA through promoting its degradation [12], [13]. Taken together, these studies imply that CCCH zinc finger proteins may be critical regulators in immunity and inflammatory response.

To gain an overall insight into the entire CCCH zinc finger gene family and to evaluate their potential role in macrophage activation, we first performed a genome-wide survey of CCCH zinc finger genes in mouse and human. 58 CCCH zinc finger genes total in mouse and 55 in human were identified and most of them have not been reported previously. Phylogenetic analysis revealed that the mouse CCCH zinc finger family was divided into 6 groups. Meanwhile, we employed quantitative real-time PCR (QPCR) to profile their tissue expression patterns in adult mice. Cluster analysis showed that most of CCCH genes were broadly expressed in all of tissues examined with various levels. Interestingly, several CCCH genes were enriched in macrophage-related organs such as thymus, spleen, lung, intestine and adipose. Consistently, a comprehensive assessment of changes in expression of the 58 members of the mouse CCCH zinc finger family during macrophage activation also revealed that these CCCH zinc finger genes were associated with the activation of bone marrow-derived macrophages by lipopolysaccharide (LPS). This study not only identified a functional module of CCCH zinc finger genes in the regulation of macrophage activation but also provided the framework for future studies to dissect the function of this emerging gene family.

## Results

### Identification of CCCH zinc finger gene family in mouse and human

The previous research revealed that CCCH zinc finger protein contained 1–6 copies of CCCH-type zinc finger motifs characterized by three Cys and one His. Berg et al. defined that the CCCH family is a group of zinc-finger protein consisting of canonical C-X_6–14_-C-X_4–5_-C-X_3_-H motif [14]. After analysis of entire CCCH family in Arabidopsis and rice, Wang et al. re-defined that the CCCH proteins are characterized by one to six C-X_4–15_-C-X_4–6_-C-X_3_-H motifs which are glycine-rich and phenylalanine-rich sequences [15]. To uncover the entire CCCH family in mouse and human, we first searched several database including EMBL-EBI, Pfam and SMART using the terms “CCCH-type zinc finger” or “C3H-type zinc finger”. Then, we used the CCCH motif sequences from defined CCCH proteins as our query to BLAST the non-redundant protein database at GenBank. After carefully checked the sequences and removed the redundancies, a total of 58 mouse genes encoded CCCH zinc finger containing proteins were identified and listed in Table 1. 55 human counterparts were also identified (data not shown). Three CCCH genes BC003883, BC019429 and Zfp36l3 were only found in murine genome, but not human. Out of the 58 mouse CCCH zinc finger proteins, 32 proteins were totally unknown. Among the other 26 known proteins, 20 proteins have been reported to be involved in RNA metabolisms including pre-mRNA splicing, mRNA transportation, subcellular localization, and stability/degradation, such as TTP family, CPSF4 family, muscleblind like family and U2af family etc [5], [16]–[18]. The other six CCCH proteins were involved in transcription, ubiquitination, and poly ADP-ribosylation [19]–[22].

**Table 1:** The Mouse CCCH Zinc Finger Family.

Gene Name	Description or Other Name	Access Number	Putative Function	Chromosome
BC003883	Hypothetical protein LOC66462	NM_001039663	Unknown	Unknown
BC019429	Putative uncharacterized protein	BC019429	Unknown	Unknown
Cpsf4	Cleavage and polyadenylation specific factor 4	NM_178576	mRNA splicing	5
Cpsf4l	Cleavage and polyadenylation specific factor 4 like	NM_026682	Unknown	11
Dhx57	Deah box protein 57	NM_198942	RNA processing	17
Dus31	Dihydrouridine synthase 3-like	NM_144858	tRNA modification	17
Helz	Helicase with zinc finger domain	NM_198298	RNA processing	11
Leng9	Leukocyte receptor cluster member 9	NM_175529	Unknown	7
Mbnl1	Muscleblind like 1	NM_020007	mRNA splicing	3
Mbnl2	Muscleblind like 2	NM_175341	mRNA splicing	14
Mbnl3	Muscleblind like 3	NM_134163	mRNA splicing	X
Mkrn1	Makorin, ring finger protein 1	NM_018810	E3 ubiquitin ligase	6
Mkrn2	Makorin, ring finger protein 2	NM_023290	Unknown	6
Mkrn3	Makorin, ring finger protein 3	NM_011746	Unknown	7
Nhn1	Conserved nuclear protein Nhn1	NM_001029993	Unknown	8
Nupl2	Nucleoporin like 2, CG1	NM_153092	mRNA exporting	5
Ppp1r10	Protein phosphatase 1, regulatory subunit 10	NM_175934	Unknown	17
Prr3	Proline rich protein 3	NM_145487	Unknown	17
Rbm22	RNA binding motif protein 22	NM_025776	Unknown	18
Rbm26	RNA binding motif protein 26	NM_134077	Unknown	14
Rbm27	RNA binding motif protein 27	NM_054080	Unknown	18
Rc3h1	Roquin, Ring CCCH domain 1	NM_001024952	mRNA destability	1
Rc3h2	Ring CCCH domain 2	AK053071	Unknown	2
Rnf113a1	Ring finger protein 113A1	NM_153503	Unknown	X
Rnf113a2	Ring finger protein 113A2	NM_025525	Unknown	12
Tiparp	TCDD-inducible PARP	NM_178892	Poly ADP-ribosylation	3
Toe1	Target of Egr-1, member 1	NM_026654	Unknown	14
Trmt1	TRM1 tRNA methyltransferase 1	NM_198020	tRNA modification	8
Unkl	Unkempt-like	NM_028789	Unknown	17
U2af1	U2 small nuclear RNP auxiliary factor, 35 kd subunit	NM_024187	mRNA splicing	17
U2af1l4	U2 small nuclear RNP auxiliary factor 1-like 4	NM_170760	mRNA splicing	7
Zc3hav1l	Zinc finger CCCH-type, antiviral 1-like	NM_172467	Unknown	6
Zc3h1	Poly (ADP-ribose) polymerase 12	NM_172893	Poly ADP-ribosylation	6
Zc3h2	Zinc finger CCCH-type, antiviral 1	NM_028864	mRNA destability	6
Zc3h3	Smad-interacting CPSF-like factor	NM_172121	mRNA splicing	15
Zc3h4	Zinc finger CCCH containing 4	NM_198631	Unknown	7
Zc3h5	Unkempt homolog	NM_172569	Unknown	11
Zc3h6	Zinc finger CCCH containing 6	NM_178404	Unknown	2
Zc3h7a	HSPC055	NM_145931	Unknown	16
Zc3h7b	Scrapie responsive gene 3	NM_001081016	Unknown	15
Zc3h8	Fetal liver zinc-finger protein 1	NM_020594	Transcription regulation	2
Zc3h9	Zgpat, Lime1	NM_144894	Unknown	2
Zc3h10	Zinc finger CCCH containing 10	NM_134003	Unknown	10
Zc3h11a	Zinc finger CCCH containing 11a	AK122341	Unknown	1
Zc3h12a	MCP-1 induced protein 1	NM_153159	Transcription regulation	4
Zc3h12b	MCP-1 induced protein 2	NM_001034907	Unknown	X
Zc3h12c	MCP-1 induced protein 3	AK082241	Unknown	9
Zc3h12d	MCP-1 induced protein 4	NM_172785	Unknown	10
Zc3h13	Zinc finger CCCH containing 13	NM_026083	Unknown	14
Zc3h14	Zinc finger CCCH containing 14	NM_029334	Unknown	12
Zc3h15	Zinc finger CCCH containing 15	NM_026934	Unknown	2
Zfp36	Tristetraproline, Nup475, Tis11	NM_011756	mRNA destability	7
Zfp36l1	Tis11b, Butyrate response factor 1	NM_007564	mRNA destability	12
Zfp36l2	Tis11d, Butyrate response factor 2	NM_001001806	mRNA destability	17
Zfp36l3	Zinc finger protein 36-like 3	NM_001009549	mRNA destability	X
Zmat5	Zinc finger, matrin type 5	NM_026015	Unknown	11
Zrsr1	U2af1-related sequence 1	NM_011663	mRNA splicing	11
Zrsr2	U2af1-related sequence 2	NM_178794	mRNA splicing	X

### Phylogenetic analysis and classification of mouse CCCH zinc finger proteins

**Figure 1:**
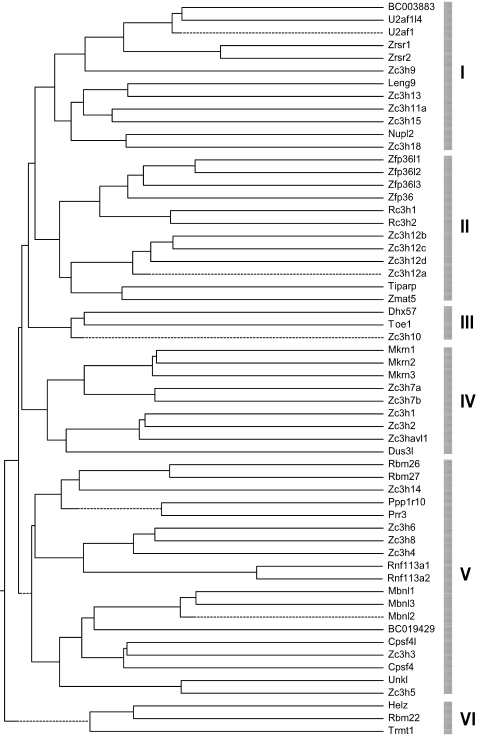
Phylogenetic analysis of 58 mouse CCCH genes. The unrooted neighbor-joining tree was constructed based on the alignment of the full-length amino acid sequences of 58 mouse CCCH proteins using ClustalW from DNASTAR. The mouse CCCH family was mainly divided into 6 groups based on their evolutionary relationship, which was denoted by the grey vertical bars on the right of the figure. The proteins are named according to their gene name (see Table 1).

To evaluate the evolutionary relationships within the mouse CCCH gene family, we performed a phylogenetic analysis by using the software of “MegAlign” from DNASTAR Inc. As shown by the neighbor-joining tree that was constructed based on the alignment of the amino acid sequences of the CCCH proteins, the mouse CCCH zinc finger family can be divided into six groups (Fig. 1). When using different method (e.g. MEGA4.0) to construct the tree, similar results were consistently reproduced. While many proteins belonged to same subfamily were clustered into same groups as expected, other proteins, previously not recognized as same subfamily, were clustered together, suggesting that they may belong to same subfamily. For example, BC003883 may form a subfamily with U2af1 and U2af1l4. Zc3h3 may be belonged to Cpsf4 subfamily. Zc3h4, Zc3h6 and Zc3h8 may form another subfamily. Interestingly, Zc3h12 family was clustered into same group with Zfp36 family and Rc3h family, suggesting that they may share similar functional features. In fact, they are all involved in the regulation of immunity and macrophage inflammation.

**Figure 2:**
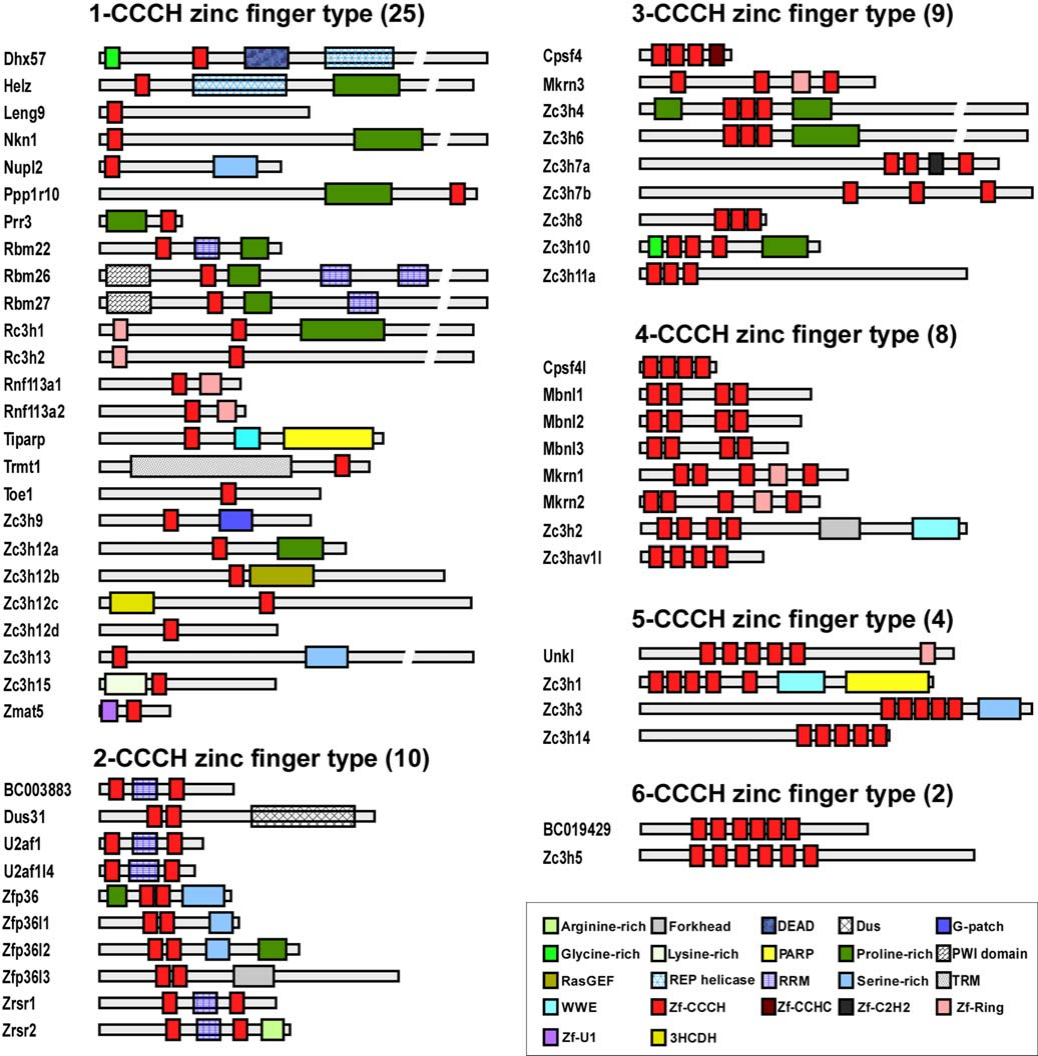
Schematic structures of 58 mouse CCCH proteins. The figure is schematic structures of 58 CCCH zinc finger proteins identified in mouse. The CCCH zinc fingers are shown by red boxes. The proteins are grouped according to the number of CCCH zinc finger. The other conserved domains are also indicated by different boxes denoted at the right-bottom corner.

In addition to contain 1–6 copies of CCCH zinc fingers, it is worth to note that most of these CCCH zinc finger proteins also carry a series of other functional domains, which are expected to direct the CCCH proteins to certain complexes and to mediate some specific activities (Fig. 2). For example, Rbm22, Rbm26, Rbm27, BC003883, U2af1, U2af1l4, Zrsr1 and Zrsr2 contain 1–2 RNA-recognized motifs, suggesting that they may bind to RNA and involve in RNA processing. Indeed, U2af1 is a small subunit of the U2 small nuclear ribonucleoprotein auxiliary factor and play important role in the regulation of pre-mRNA splicing [17]. Many CCCH proteins such as Helz, Nkn1, Ppp1r10, Prr3, Rbm22, Rbm26, Rbm27, Rc3h1, Zc3h12a, Zfp36, Zfp36l2, Zc3h4, Zc3h6 and Zc3h10 contain 1–2 proline-rich domains. Proline-rich domain usually mediates protein-protein interaction, suggesting that those proteins may form a complex with other proteins and performed distinct functions. In consistent with the phylogenetic analysis above, the proteins in same family displayed similar domain architecture (Fig. 2). For example, Zc3h12 family members all contain a single CCCH zinc finger motif at the middle region. Rc3h1 and Rc3h2 contain a single CCCH motif at the middle region and a ring-finger at N-terminal. In addition, the evidence that BC003883 has much similar domain architecture with U2af1 and U2af1l4 further supports the family prediction based on the phylogenetic analysis above.

### The analysis and classification of CCCH zinc finger motifs

**Figure 3:**
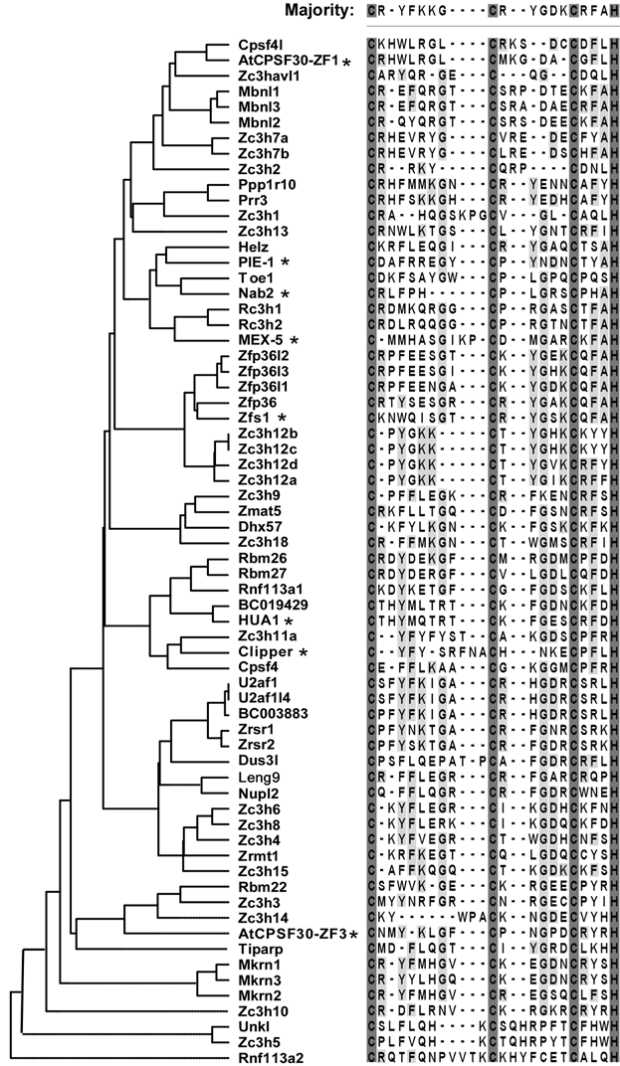
Phylogenetic analysis of CCCH motifs. The unrooted neighbor-joining tree was constructed based on the alignment of the consensus sequences of CCCH zinc finger motifs from 58 mouse CCCH proteins using ClustalV from DNASTAR. For two or multiple copies of CCCH-zinc finger containing proteins, the first zinc finger sequence was selected to analyze. Several well-studied CCCH proteins from other species were also analyzed together, which were marked with asterisk (*). The sequence alignment was also shown at the right. Three cystine and one histine residues were complete conserved and marked with a grey background. The other conserved residues were also marked by a light grey background and represented on the top.

The CCCH zinc finger motif is a highly conserved motif, which has been found in plant, invertebrate and vertebrate. As shown in Fig. 2, 25 CCCH proteins contain single CCCH motif. The other 33 CCCH proteins contain two or multiple CCCH motifs. To further determine the sequence features of these CCCH motifs, we performed phylogenetic analysis based on the alignment of the amino acid sequences of the CCCH zinc finger motifs. Since the multiple motifs in same protein are usually similar and have redundant function, the first CCCH motif in these proteins was selected for analysis. In addition, several well-studied CCCH motifs from other species such as HUA1 and AtCPSF30 (from plant), NAB2 and Zfs1 (from yeast), MEX-5 and PIE-1 (from worm) and Clipper (from fly) were also imported. As shown in Fig. 3, the tree topology, as well as the subfamily organization, was much similar to that constructed from the full-length CCCH proteins (Fig. 1). As expected, the proteins within same subfamily exhibit the common CCCH motifs, suggesting the major functional role of CCCH motif in these proteins. Consistently, BC003883 shared a similar CCCH motif with U2af1 and U2af1l4, and Zc3h4, Zc3h6 and Zc3h8 were clustered into one subfamily, further supporting that they may be from same subfamily and may have similar function. Interestingly, three putative orthologs (BC019429 and HUA1, Helz and PIE-1, Zc3h11a and Clipper) were identified in the tree. HUA1 is a CCCH protein from plant, which directly binds to mRNA and promotes mRNA degradation [23]. PIE-1 is a CCCH protein from *Caenorhabditis elegans* and regulates cell fate determination [24]. Clipper, a *Drosophila melanogaster* protein, contains five copies CCCH motifs and functions directly in RNA metabolism [25]. It would be interesting to observe the function of BC019429, Helz and Zc3h11a.

CCCH motif is a small functional unit containing 17–25 amino acids. As shown in Fig. 3, three cysteins and one histidine residues were completely conserved in all CCCH motifs. However, the space and amino acids between CCCH residues varies, which may determine their substrate specificity. It is worthy to note that 79% CCCH motifs analyzed are C-X_7–8_-C-X_5_-C-X_3_-H type, suggesting that C-X_7–8_-C-X_5_-C-X_3_-H motif may be an ancestor of other CCCH motifs. Beside the CCCH residues, glycine and phenylalanine were also highly conserved.

### Expression profiling of the CCCH zinc finger family in mouse tissues

Since gene expression patterns can provide important clues for gene function, next we used QPCR to survey the expression of entire CCCH family in 16 tissues from adult C57/BL6J mice. We have compared the expression patterns of several CCCH genes with the previously reported data and found that they were very similar, suggesting that our data are convincing. For example, Mbnl1 and Mbnl2 showed high expression levels in skeletal muscle, which is consistent with previous reports and matches with their important function in muscle differentiation [26], [27].

**Figure 4:**
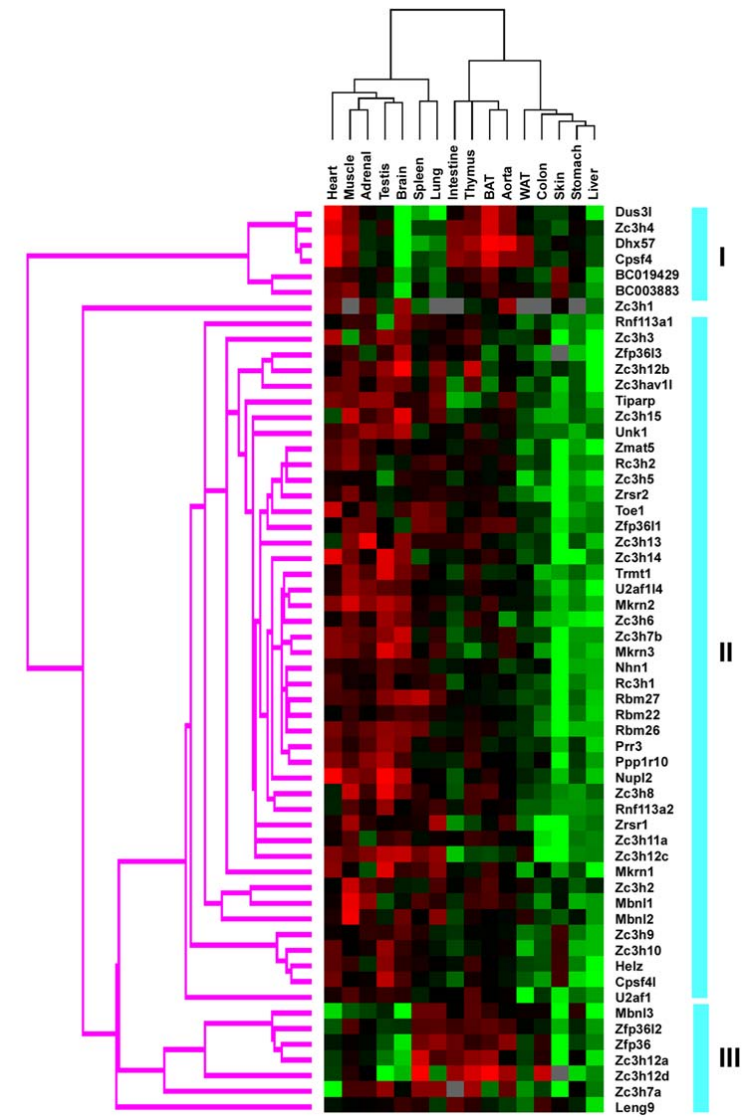
Hierarchical clustering of mouse CCCH family relative to tissue expression pattern. The mRNA tissue-distribution profile of the mouse CCCH family in the C57/BL6 mouse was evaluated by hierarchical clustering using Cluster2.11 software as described in “Materials and Methods”. The CCCH family was mainly divided into three groups based on their tissue expression patterns and indicated by the blue vertical bars on the right of the figure. Red is represented as high expression level, whereas green means low expression level.

To study the potential relationship between gene expression, function and physiology, the QPCR results were analyzed by clustering analysis. As shown in Fig. 4, the accumulation of CCCH gene transcripts not only is associated with different tissues, but also the expression pattern of each CCCH gene member differed. According to the expression profiles, CCCH genes can be classified into three groups. The largest group including 44 CCCH genes was broadly expressed in more than half but not all tissues with various expression levels. The other two groups were much smaller, but displayed distinct distribution patterns. As shown in Fig. 4, one group containing 6 genes Dus3l, Zc3h4, Dhx57, Cpsf4, BC019429 and BC003883 showed highly expression levels in high energy expense tissues such as heart, muscle, aorta and brown adipose tissue, suggesting that they may involve in the regulation of fatty acid metabolism. The third group including Mbnl3, Zfp36l2, Zfp36, Zc3h12a, Zc3h12d, Zc3h7a and Leng9 was highly enriched in macrophage-related organs such as spleen, lung, intestine, thymus, aorta and adipose tisuue, suggesting that they may involve in the regulation of innate immunity and inflammatory response. Overall, the CCCH family members showed diverse expression patterns. The genes with specific expression patterns can be the focus of functional studies for their possible roles in specific tissues. The entire expressed data set is available in Supplemental Table S2.

### Expression changes of the CCCH family during macrophage activation

Activated macrophages play an important role in many inflammatory diseases. However, the molecular mechanisms controlling macrophage activation are not completely understood. TTP (also known as Zfp36) is a well-studied CCCH zinc finger protein with its unique role in limiting macrophage inflammatory response by promoting TNFα mRNA degradation [5]. Another CCCH protein Zc3h12a also regulated macrophage activation by a distinct mechanism [4]. Given the importance of this limited number of CCCH proteins in controlling macrophage function, we sought to identify the full complement of the CCCH zinc finger family with macrophage lineage. Here we further employed QPCR to provide a comprehensive assessment of changes in expression of the 58 members of mouse CCCH-zinc finger gene family during LPS-stimulated macrophage activation. The entire expressed data set was attached into Supplemental Table S3.

**Figure 5:**
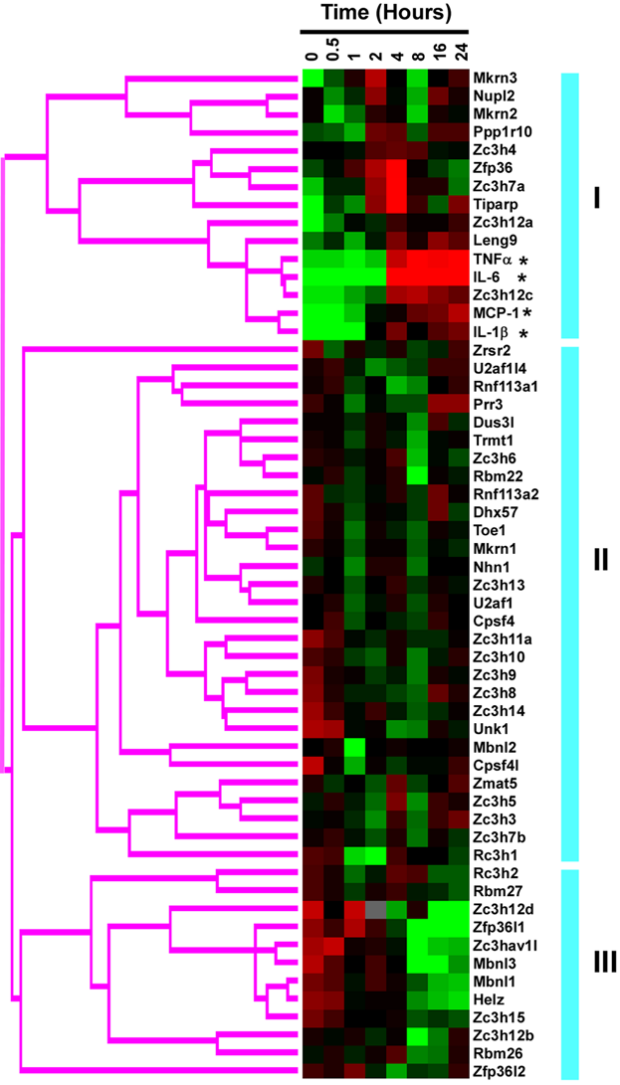
Hierarchical clustering of mouse CCCH family relative to dynamic expression profile during macrophage activation. The primary macrophages were stimulated with LPS for different times as indicated on the top. The mRNA expression profile of the mouse CCCH family during macrophage activation was evaluated by hierarchical clustering using Cluster2.11 software as described in “Materials and Methods”. The CCCH family was mainly divided into three groups based on their dynamic expression patterns and indicated by the blue vertical bars on the right of the figure. Red is represented as high expression level, whereas green means low expression level. Several markers of macrophage activation were also analyzed together and marked with asterisk (*).

**Figure 6:**
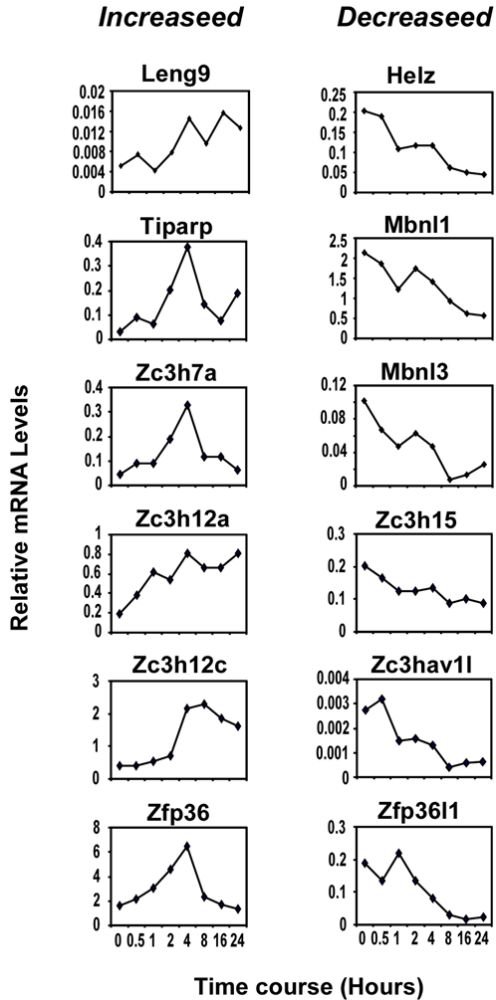
Dynamic expression changes of CCCH zinc finger genes during macrophage activation. Represented CCCH genes with dynamic expression changes during LPS-induced macrophage activation were shown. The mRNA levels were calculated according to 2^−ΔCt^ and plotted across the time course.

Except 6 CCCH genes (BC003883, BC019429, Zc3h1, Zc3h2, Zfp36l3 and Zrsr1) undetected in the macrophages across the whole time course, the other genes were expressed with distinct dynamic patterns. According to the expression profiles, CCCH genes can be classified into three groups (Fig. 5). The largest group including 29 CCCH genes was constantly expressed during the time course of macrophage activation. Interestingly, 11 members of CCCH proteins were significantly increased during macrophage activation with distinct patterns. Mkrn3, Nupl2, Mkrn2 and Ppp1r10 obtained first peak at 2 hours, then declined to basal level at 8 hours, but increased again at 16 hours of stimulation. Zc3h4, Zfp36, Zc3h7a and Tiparp were transient induced within 4 hours and then declined in the remained time. Remarkably, Zc3h12a, Zc3h12c and Leng9 shared similar expression patterns with several macrophage activation markers such as TNFα, MCP-1, IL-1β and IL-6, which dramatically increased at 4 hours and sustained at least for 24 hours. In contrast with these genes, 12 CCCH genes Rc3h2, Rbm27, Zc3h12d, Zfp36l1, Zc3hav1l, Mbnl3, Mbnl1, Helz, Zc3h15, Zc3h12b, Rbm26 and Zfp36l2 were significantly down-regulated during macrophage activation. The more quantitative figures of several genes were shown in Fig. 6. The CCCH genes with dynamic expression changes across the time course of macrophage activation are strongly implicated in the regulation of the critical process.

## Discussion

Zinc finger proteins comprise a large superfamily and are important regulators of cellular processes. It is estimated that 1% of all mammalian genes encoded zinc fingers [28]. There are at least 14 different types of fingers, categorized by the nature and spacing of their zinc-chelating residues. Most of zinc fingers presented in transcription factors are CCHH- or CCCC-type [29]. The CCCH-type zinc finger is less common compared to CCHH- or CCCC-type zinc fingers and represents approximately 0.8% of all zinc fingers [30], [31]. The objective of this study is to comprehensively and non-redundantly identify all of mouse and human CCCH zinc finger genes on a whole-genome scale, and subsequently to obtain a global view of this gene family in the context of macrophage activation. As a consequence of whole genome searching, totally 58 CCCH genes were identified in mouse and 55 in human. The family size of human and mouse CCCH genes is smaller than the size of CCCH gene family in Arabidopsis and rice, which contain 68 and 67 members respectively. Although most of CCCH proteins are less characterized, some CCCH proteins play important roles in many cellular processes especially RNA metabolism. For example, Cpsf4 and U2af1 are important components in the protein complex controlling pre-mRNA splicing [16], [18]. Muscleblind like protein family is essential for muscle and eye differentiation through controlling proper pre-mRNA splicing of several important genes including skeletal muscle chloride channels, cardiac troponin T and insulin receptor [26]. Three CCCH genes BC003883, BC019429 and Zfp36l3 were only found in murine genome, but not human. In addition, ZC3H11B does not exist in mouse, but exist in human as a pseudogene and not analyzed in this study.

Based on the amino acid sequence alignment, the whole CCCH gene family can be divided into 6 groups. Some subfamilies were clustered into same groups, suggesting that they may have some functional association. For example, Zc3h12 family was consistently to cluster into same group with Zfp36 family by full-length protein sequences or CCCH motif sequences, strongly suggesting these two families may share some similar functional features. Indeed, both Zfp36 and Zc3h12a are important regulators in macrophage inflammation [4], [5]. It is well-known that Zfp36 can bind to ARE elements in the 3′UTR of TNFα mRNA and promote its degradation, by which it feedback controls macrophage inflammation [5]. Interestingly, our recent work showed that Zc3h12a also feedback regulated macrophage inflammation by a distinct mechanism. Zc3h12a did not promote TNFα mRNA degradation, but strongly repressed its transcription [4], Liang and Fu, unpublished data. The other interesting finding is several new subfamilies have been identified by clustering analysis and domain architecture assembly. BC003883 may be a new member of U2af1 family and also functions as a splicing factor. Zc3h1 may belong to same subfamily with Zc3h2 (also known as ZAP) and Zc3hav1l. As ZAP is a host antiviral factor that specifically inhibits the replication of some virus by preventing accumulation of the virus mRNA in the cytoplasm [10], it would be interesting to find out the function of Zc3h1 and Zc3hav1l.

As we shown in this study, CCCH motif is a highly conserved functional unit. The question is what CCCH motif really does? Traditionally, zinc finger proteins are regarded as DNA-binding proteins. Recent studies demonstrated that zinc fingers can also mediate interaction to RNA, protein and even lipids. However, emerging evidence revealed that, as an unusual family of zinc finger proteins, the CCCH-type zinc finger proteins mainly bind to RNA and, therefore, facilitate their metabolism or processing [30], [31]. Indeed, the structure analysis revealed that each CCCH zinc finger module from Zfp36l1 binds to the sequence UAUU; thus, the tandem zinc fingers bind to UAUU-UAUU. This sequence is remarkably similar to the nonamer (UUAUUUAUU) that was identified as the functional ARE motif in biochemical studies [32]. These studies suggest that CCCH-zinc finger may be a novel RNA-binding motif. However, there is also evidence that some CCCH zinc finger motifs bind to DNA or proteins. For example, Zc3h8 (also known as fetal liver zinc finger protein 1, Fliz1) directly binds to a regulatory element located within the first intron of GATA-3 gene through its CCCH motifs and strongly represses GATA-3 transcription [19]. Niu et al. used chromosome immunoprecipitation analysis demonstrate that Zc3h12a (also known as Mcpip) binds to the promoters of cadherin 12 and cadherin 19 [22]. The Arabidopsis CPSF30 ortholog, AtCPSF30, possesses an unexpected endonucleolytic activity to selectively degrade RNA, which also contains three copies of CCCH motifs. Surprisingly, mutation in the first CCCH motif of the protein abolish RNA binding by AtCPSF30 but have no discernible effects on nuclease activity. In contrast, mutations in the third CCCH motif eliminate nuclease activity of the protein, suggesting that even in the same protein different CCCH motifs may play different but coordinative roles [33]. Taken together, CCCH motif primarily mediates RNA binding and involves in RNA metabolism. However, it may also bind to DNA or proteins and exert transcriptional regulation or specific enzymatic activity.

To further understand the function of the whole CCCH gene family, we utilized QPCR to profile their expression patterns in 16 adult mouse tissues. The advantage of using QPCR-expression profiling of selected gene arrays to study complex physiologic as well as pathophysiologic processes was recognized previously [34]–[36]. Cluster analysis revealed that most of CCCH genes were broadly expressed in almost all tissues examined with various expression levels. Interestingly to note that several CCCH genes including Zc3h12a, Zc3h12b, Zc3h12c, Zc3hav1l, Tiparp and Zc3h7a were clustered in same group, which were highly enriched in macrophage-related organs such as spleen, thymus, lung, intestine and colon. To further evaluate the potential involvement of the entire CCCH family in macrophage activation, we used QPCR to assess their dynamic expression changes during LPS-induced macrophage activation. Although most of CCCH genes were expressed in macrophages, 23 CCCH genes showed dynamic expression changes during the time course of LPS-stimulated macrophage activation. Interestingly, the group enriched in macrophage-related organs including Zc3h12a, Zc3h12d, Zfp36l2, Zc3h7a, leng9 and Mbnl3 displayed dynamic expression patterns during macrophage activation, strongly suggesting that they may play important role in the regulation of macrophage activation.

The extremely interesting family is Zc3h12 family, which was clustered into same group with Zfp36 family based on the amino acid alignment of both full-length proteins and CCCH motifs. Zc3h12a (also designated as MCPIP) was significantly increased in ischemic heart and promoted cell apoptosis, and mediated MCP-1 induced angiogenesis, suggesting that it may be an important player in human ischemic heart disease [3], [22]. Our recent studies also demonstrated that at least one member of this family Zc3h12a strongly inhibited macrophage activation by repressing NF-κB signaling pathway [4], Liang and Fu, unpublished data. However, how it exerts this action or does any RNA component involve in this process is under investigation. Recent report showed that overexpression of Zc3h12D (also designated as p34) exerts tumor suppressor function and an A/G SNP of Zc3h12D was associated with lung cancer [37]. Considering NF-κB signaling is over-activated in most of cancer, it would be extremely interesting to observe whether Zc3h12D is also a negative regulator of NF-κB signaling.

In summary, we have identified the whole CCCH gene family. Through unbiased clustering analysis, several new subfamilies previously not recognized were discovered. The quantitative expression profiles of whole gene family both in normal mouse tissues and across the course of macrophage activation not only help to identify a functional module of CCCH zinc finger genes in the regulation of macrophage activation but also provide the framework for future studies to dissect the function of this emerging family.

## Materials and Methods

### Identification of CCCH-zinc finger protein family in mouse and human

To identify the entire CCCH protein family across whole genome, we first searched several protein databases EMBL-EBI, SMART, and Pfam using the terms “CCCH-type zinc finger” or “C3H-type zinc finger”. Then, we used the CCCH motif sequences from the defined CCCH proteins as our query to BLAST the non-redundant protein database at GenBank (www.ncbi.nlm.nih). Totally 128 protein sequences containing at least 1 CCCH zinc finger (C-X_4–15_-C-X_4–7_-C-X_3_-H motif) were identified. After carefully check the sequences and removed the redundancies, 58 CCCH proteins in mouse and 55 in human were identified.

### Alignment and phylogenetic analysis of CCCH proteins

Multiple alignments of amino acid sequences of full-length CCCH proteins or CCCH motifs were performed using ClustalW or ClustalV from DNASTAR and then generated the phylogenetic tree by the neighbor-joining algorithm. We used an additional program MEGA4.0 to confirm the results.

### Mice and tissue harvest

Six-week-old male pure strain C57/Bl6J mice were purchased from Jackson Laboratory (Bar Harbor, ME). Mice were allowed to reach 8–9 weeks of age on a 12 hr light/dark cycle and fed standard diets. The mice (n = 6) at 8–9 weeks of age were sacrificed by halothane inhalation (Halocarbon Laboratories; River Edge, NJ). Whole tissues were collected and snap frozen in liquid nitrogen. Tissues were isolated from appropriate anatomical locations according to established laboratory methods. Brown adipose was collected from the dorsal interscapular depression and any surrounding white fat or connective tissue was removed. White adipose was collected from the epididymal area. Skeletal muscle (quadriceps) was isolated from both femurs. Dorsal skin was shaved prior to collection. All protocols were approved by the University of Central Florida Institutional Animal Care and Use Committee.

### Cells

Mouse bone marrow-derived macrophages (BMDM) were generated from bone marrow stem cells obtained from femurs of the male C57BL/6 mice (2–4 month old). After lysis of the red blood cells, 4×10^6^ of bone marrow stem cells were inoculated in 6-well plate with complete Dulbecco's modified Eagle's medium containing 10% fetal bovine serum, 30% L929 conditional medium, 100 units/ml streptomycin, and 100 units/ml penicillin [4]. After 5-d culture, the fully differentiated and matured BMDM were quiescent for 24 hours by cultured in macrophage serum-free medium and then treated with 1 µg/ml LPS (*Escherichia coli* 026:B6-derived, Sigma) for different time points.

### RNA isolation and cDNA preparation

Total RNA was isolated from mouse tissues or cells using RNA STAT-60 reagent (Tel-Test, Friendswood, TX) followed the manufacturer's instruction. Total RNA was pooled in equal quantities for each tissue (n = 6). Genomic DNA contamination was eliminated by DNase treatment using Ambion's Turbo DNA-*free* kit (Austin, TX). Preparation of cDNA for QPCR assays was performed using a commercial available kit (Invitrogen) followed the manufacturer's protocol.

### QPCR and clustering analysis

QPCR was performed with iCycler Thermal Cycler (Bio-Rad, Herculer, CA) using 2×SYBR Green master mix (Bio-Rad). 50 cycles were conducted as follows: 95°C for 30 s, 60°C for 30 s, preceded by 1 min at 95°C for polymerase activation. All primers for the 58 CCCH genes were validated using universal cDNA standards (BD Clontech).The primer sequences for all CCCH genes were summarized in Table S1. Quantification was performed by ΔCt method, with cyclophilin used for normalization. mRNAs with cycle times ≥34 were determined to be undetected. Normalized mRNA levels are expressed as arbitrary units by transformed the cycle times using 2^−ΔCt^. Hierarchical clustering analysis was performed on the normalized, log- transformed and median-centered RNA levels by calculating Pearson correlation as distance followed by average linkage analysis using Cluster2.11 software. The resulting cluster analysis was then displayed as a tree using TreeView1.60 software.

## Supporting Information

Table S1 (0.09 MB DOC)

Table S2 (0.21 MB DOC)

Table S3 (0.13 MB DOC)
