# Supplementary material for: Genome-Wide Survey and Expression Profiling of CCCH-Zinc Finger Family Reveals a Functional Module in Macrophage Activation
Source: PLoS One. 2008 Aug 6;3(8):e2880. doi: 10.1371/journal.pone.0002880 (PMC2478707; doi:10.1371/journal.pone.0002880)
Supplement: Table S1 — (0.09 MB DOC) [file pone.0002880.s001.doc]

**Table S1. QPCR primers for mouse CCCH gene family**

| **Gene Name** | **Forward** | **Reverse** |
| --- | --- | --- |
| BC003883 | GGGAGCTTAGGCGACAGTTG | TGCTTGAGGACTGCGACTTCT |
| BC019429 | CCCTCTACAAGATCCGCCATT | AAGGTAGCGGGAAGAGCTTAGAA |
| Cpsf4 | GGAAGACGGCTGGCAGTTC | ATGATCCACCCGTCCTCTTTAG |
| Cpsf4l | TTGAGCTGGATGTGGAATCG | GACTTGTCCATGCCCTGGAA |
| Dhx57 | CCCCGCTGGGTTATCACTT | CGGAAGATAGAACCTAAGAGCATCA |
| Dus3l | CCATCGACCTCGTCTACAAGAAG | CCGCACAATCTGTTGGAACTT |
| Helz | GGTTTATTGCCCTGTGTCATGAA | CCTCTAACTGTGCCTTGATCTGTTC |
| Leng9 | CTGACTTTTCGGTGGGCTACA | GGCGCAGAATGGCTCTTC |
| Mbnl1 | GATTTTTCAGCTGGAAAGATTTGC | GCAGATTTGGCCCAATGG |
| Mbnl2 | CCCAACAGAGGTTCTACCCACTA | TGAGCCCGGGACAGTGA |
| Mbnl3 | TTCCAACAACTACAGGCAACCA | ACACGGAGTTTCTTGTTGAGAGATT |
| Mkrn1 | CAGCGAGCGTCGCTTTG | CACTTGCGAATACACTTGAGACAGT |
| Mkrn2 | CAGAGATGAAGCCCCATTCC | GCTGCTGGCCTCCAAGTC |
| Mkrn3 | CGTGCTGTTTAAAAGCCGTAAA | CTCTCAGGCAAAGCAACTTCTTAAG |
| Nhn1 | GCACGAAGACGTCGCAAAA | GAGTAGGATGAAGACCGTGACGAT |
| Nupl2 | GAGTCCCTCTGGCTCTACAACAG | GTGGGCTAGGACTGCCAAAA |
| Ppp1r10 | CCGTGAGACCAGCTTTCATTAA | CACTCCCAAAGGCAGTGCAT |
| Prr3 | CTTTCACGTAAAACCCCAGTGTT | TCGACCTCAAGGCCAGACA |
| Rbm22 | CCCGGGACTCCCGATATG | CGCGTCCTCCCAGTTCTG |
| Rbm26 | CCGAACCGTTCCCATTGAG | GGGATTAGGTGCTTGGTTGGT |
| Rbm27 | ACTGGCTTCCCTCCCCTTAA | TTACAATGTAGGAAAAGACCTTCAGCTA |
| Rc3h1 | CACAATGGACGGATTTCCTTTC | TGATGGGCTTTCGAATTGTTT |
| Rc3h2 | GCAACTAAGCCCATCAGCGTAT | ATGTAGCATCGTTGCCATATGAA |
| Rnf113a1 | GCGCTAAACAGAAGGCAACCT | GAGACCCTCCGGTTCATTCTC |
| Rnf113a2 | GGTTGTGCGACCGGAGAA | TACCGCTGCCACTCGTCTTC |
| Tiparp | AGCCATATCTGCTGAGTTGAATTG | CCAGTGCATCTGGCAAAGC |
| Toe1 | GCCATCACTCTTGCTAGCCTTAA | TCCCCGAGCCCACTCA |
| Trmt1 | GCGTTGCTGGGAAAAGGAA | GGAATGCTGGACTGCTTTCAG |
| Unkl | CCGCGCTTTGGAACGA | CACTAAGACGAAGATCTAAGGCAGAA |
| U2af1 | GCTGTCCCCAGTAACTGACTTCA | GCCCCCTCTTGTGCACTCT |
| U2af1l4 | GGCATGGTCGCTTCTGAGA | AAGGGACGCTGGACAGGAA |
| Zc3hav1l | CCACATGCTGGGCAAGTG | GTGTGTGGATATCATGGGACAGA |
| Zc3h1 | GCTGTGTGGTGCCATCGA | TGCACTTCCTTGGGCAATG |
| Zc3h2 | CCAGAGACCCTGCGATAACC | CCGCTCGGACTGCGAATA |
| Zc3h3 | CCGGCGCATCCCATTAT | CCCTCCCCCAGTGATTGC |
| Zc3h4 | TGCAGGCTGCCTCTTTTATATTG | TGTGTGCGTCAGGTCTCCTTAT |
| Zc3h5 | GGACACTGAGCGCAGGTATCA | TGGTGCAGTTGCCTTTTGAG |
| Zc3h6 | CCTCCCATTGCTCGTGACA | CAGACAGAGGGCAGTGACTTGT |
| Zc3h7a | TCTGCTTGATTGGCATCTATCG | GGCCTGTCAAACAGCCTTTTT |

Table S1. Continued

| **Gene Name** | **Forward** | **Reverse** |
| --- | --- | --- |
| Zc3h7b | CTCCAAGATCCGTCAGTTCCA | CGCGCAGGCAACCATAG |
| Zc3h8 | CACAAAATAGGCCAAGAGCAGTT | CCCCTAGCAGCTCTGTCATCA |
| Zc3h9 | CGCACTGAGGCTACAGAGTCAT | TTCCACCACTCTGGCATAGCT |
| Zc3h10 | TGAGGAACGTCTGCAAACGA | CCAAATTGGATACCTCACTCATGTC |
| Zc3h11a | TGTTGTAAGCGGCTCATCAAGA | TGGCAGTCCTGTGGCTTTCT |
| Zc3h12a | CTGCCTCTCAGTCCAGCTCT | GGAGTGAGTCCTGGGTGTGT |
| Zc3h12b | ACCCTGAGCAGATGAGCTGT | AAAATGCTGCTCTGGGAGAA |
| Zc3h12c | CATGGCTTTTGCTTGCTGTA | CAAGCCTCCAGGCTGACTAC |
| Zc3h12d | CTGAAAAGAGAGGGGCACTG | AATTCCATCTTGCTCCGATG |
| Zc3h13 | ATTCCAGAAGCCATAGCTCAAGA | AACGACCCGGACCTTGAGT |
| Zc3h14 | GCAACTTCATCCGCAGCAA | CGGATACCTGGCTGCTTTGT |
| Zc3h15 | TGGACACTGAGAATGGGACTTG | GGGAGAACTTCAAACAGCTTTAACA |
| Zfp36 | CACCACCTCCTCTCGATACAAGA | GGCCCCGTAGCGACAAC |
| Zfp36l1 | ACGACACACCAGATCCTAGTCCTT | ACACGAGGGTGGTGGTCATC |
| Zfp36l2 | GGCCGCACAAGCACAAC | CGAGACTCGAACCAAGATGAATAA |
| Zfp36l3 | CGCGGAATGGCGAACA | TCGAGCCGCTCACCTTCT |
| Zmat5 | CGAAAGAAGCACCTGAATGGA | CATCTCGGAACATGTCGTACCA |
| Zrsr1 | CCCCGCTCTGCGGTAAA | TGTATTGTTTTCGGCTGAGTTTCT |
| Zrsr2 | GGAGAAAGATCGAGCCAACTGT | GTTTACGTGAACACCTATCTCCAAATC |
