# Supplementary material for: Genome-Wide Survey and Expression Profiling of CCCH-Zinc Finger Family Reveals a Functional Module in Macrophage Activation
Source: PLoS One. 2008 Aug 6;3(8):e2880. doi: 10.1371/journal.pone.0002880 (PMC2478707; doi:10.1371/journal.pone.0002880)
Supplement: Table S2 — (0.21 MB DOC) [file pone.0002880.s002.doc]

**Table S2. Tissue Expression Profiles of the Mouse CCCH Gene Family**

| **ID** | **Adrenal** | **Aorta** | **Brain** | **BAT** | **Colon** | **Heart** | **Intestine** | **Liver** | **Lung** | **Muscle** | **Skin** | **Spleen** | **Stomach** | **Testis** | **Thymus** | **WAT** |
| --- | --- | --- | --- | --- | --- | --- | --- | --- | --- | --- | --- | --- | --- | --- | --- | --- |
| **BC003883** | 8.63E-05 | 0.000114 | 1.16E-05 | 0.000185 | 6.1E-05 | 0.000228 | 8.05E-05 | 2.31E-05 | 3.76E-05 | 0.000322 | 0.000228 | 0.000106 | 6.54E-05 | 6.54E-05 | 0.000106 | 0.000114 |
| **BC019429** | 0.000131 | 0.00014 | 2.66E-05 | 0.00037 | 6.54E-05 | 0.00028 | 0.000185 | 2.66E-05 | 4.96E-05 | 0.000228 | 0.000425 | 9.25E-05 | 0.00015 | 0.000213 | 0.000228 | 0.000106 |
| **Cpsf4** | 0.071794 | 0.406126 | 0.003645 | 0.870551 | 0.088388 | 2.143547 | 0.435275 | 0.058315 | 0.054409 | 0.329877 | 0.066986 | 0.066986 | 0.133972 | 0.108819 | 0.353553 | 0.378929 |
| **Cpsf4l** | 0.005154 | 0.003906 | 0.008974 | 0.004809 | 0.001122 | 0.012691 | 0.001822 | 0.00037 | 0.003645 | 0.004809 | 0.008373 | 0.004487 | 0.000644 | 0.025383 | 0.005524 | 0.002093 |
| **Dhx57** | 0.03125 | 0.406126 | 0.001047 | 0.757858 | 0.029157 | 0.659754 | 0.143587 | 0.022097 | 0.014579 | 0.176777 | 0.050766 | 0.009618 | 0.041235 | 0.035897 | 0.203063 | 0.143587 |
| **Dus3l** | 0.076947 | 0.203063 | 0.001202 | 0.466516 | 0.038473 | 0.812252 | 0.076947 | 0.007813 | 0.006801 | 0.189465 | 0.038473 | 0.014579 | 0.066986 | 0.054409 | 0.153893 | 0.066986 |
| **Helz** | 0.25 | 0.329877 | 0.535887 | 0.378929 | 0.189465 | 0.870551 | 0.329877 | 0.038473 | 0.378929 | 0.615572 | 0.659754 | 0.466516 | 0.116629 | 1.319508 | 0.659754 | 0.203063 |
| **Leng9** | 0.03125 | 0.017948 | 0.050766 | 0.023683 | 0.054409 | 0.025383 | 0.071794 | 0.003906 | 0.017948 | 0.029157 | 0.033493 | 0.041235 | 0.022097 | 0.033493 | 0.044194 | 0.011049 |
| **Mbnl1** | 2.462289 | 0.933033 | 0.574349 | 1.319508 | 0.176777 | 0.757858 | 0.435275 | 0.164938 | 0.933033 | 5.656854 | 0.574349 | 1.319508 | 0.133972 | 0.307786 | 1.148698 | 0.435275 |
| **Mbnl2** | 1.515717 | 0.933033 | 2.639016 | 1 | 0.615572 | 1.319508 | 0.535887 | 0.25 | 3.482202 | 9.189587 | 0.435275 | 1 | 0.870551 | 0.757858 | 1.231144 | 1.515717 |
| **Mbnl3** | 0.054409 | 0.094732 | 0.0017 | 0.153893 | 0.108819 | 0.013602 | 0.101532 | 0.008373 | 0.25 | 0.044194 | 0.002093 | 0.25 | 0.143587 | 0.071794 | 0.217638 | 0.108819 |
| **Mkrn1** | 0.25 | 0.535887 | 0.812252 | 0.933033 | 0.5 | 1.866066 | 0.466516 | 0.035897 | 1.741101 | 0.574349 | 0.125 | 0.933033 | 0.287175 | 4 | 0.406126 | 0.108819 |
| **Mkrn2** | 0.267943 | 0.066986 | 0.287175 | 0.143587 | 0.038473 | 0.25 | 0.047366 | 0.013602 | 0.116629 | 0.535887 | 0.013602 | 0.088388 | 0.023683 | 0.406126 | 0.066986 | 0.066986 |
| **Mkrn3** | 0.00296 | 0.00296 | 0.007813 | 0.001289 | 0.000425 | 0.005921 | 0.001047 | 0.000301 | 0.003401 | 0.005154 | 9.25E-05 | 0.0017 | 0.000456 | 0.013602 | 0.000488 | 0.001822 |
| **Nhn1** | 0.101532 | 0.038473 | 0.125 | 0.082469 | 0.066986 | 0.108819 | 0.0625 | 0.014579 | 0.054409 | 0.088388 | 0.003401 | 0.108819 | 0.015625 | 0.164938 | 0.076947 | 0.054409 |
| **Nupl2** | 0.076947 | 0.027205 | 0.133972 | 0.038473 | 0.015625 | 0.435275 | 0.011842 | 0.004487 | 0.029157 | 0.094732 | 0.004809 | 0.027205 | 0.014579 | 0.757858 | 0.025383 | 0.020617 |
| **Ppp1r10** | 0.143587 | 0.041235 | 0.076947 | 0.041235 | 0.044194 | 0.143587 | 0.029157 | 0.009618 | 0.033493 | 0.076947 | 0.003906 | 0.033493 | 0.025383 | 0.203063 | 0.076947 | 0.027205 |
| **Prr3** | 0.133972 | 0.058315 | 0.189465 | 0.047366 | 0.044194 | 0.164938 | 0.071794 | 0.009618 | 0.088388 | 0.088388 | 0.011842 | 0.058315 | 0.044194 | 0.25 | 0.101532 | 0.038473 |
| **Rbm22** | 0.267943 | 0.25 | 0.5 | 0.267943 | 0.094732 | 0.25 | 0.233258 | 0.038473 | 0.329877 | 0.406126 | 0.012691 | 0.466516 | 0.088388 | 0.378929 | 0.329877 | 0.176777 |
| **Rbm26** | 0.406126 | 0.116629 | 0.535887 | 0.203063 | 0.047366 | 0.5 | 0.133972 | 0.03125 | 0.125 | 0.287175 | 0.012691 | 0.435275 | 0.03125 | 0.615572 | 0.25 | 0.108819 |
| **Rbm27** | 0.094732 | 0.050766 | 0.307786 | 0.058315 | 0.038473 | 0.133972 | 0.076947 | 0.011049 | 0.153893 | 0.125 | 0.004809 | 0.378929 | 0.029157 | 0.233258 | 0.0625 | 0.033493 |
| **Rc3h1** | 0.435275 | 0.406126 | 0.406126 | 0.329877 | 0.176777 | 0.812252 | 0.176777 | 0.076947 | 0.659754 | 0.466516 | 0.025383 | 0.535887 | 0.125 | 0.933033 | 0.378929 | 0.233258 |
| **Rc3h2** | 0.574349 | 0.5 | 0.287175 | 0.307786 | 0.217638 | 0.812252 | 0.378929 | 0.094732 | 0.659754 | 1.231144 | 0.03125 | 0.535887 | 0.108819 | 0.203063 | 0.466516 | 0.164938 |
| **Rnf113a1** | 0.019237 | 0.012691 | 0.044194 | 0.010309 | 0.008373 | 0.011842 | 0.012691 | 0.002244 | 0.017948 | 0.020617 | 0.002762 | 0.020617 | 0.003906 | 0.002577 | 0.017948 | 0.003906 |
| **Rnf113a2** | 0.116629 | 0.108819 | 0.094732 | 0.101532 | 0.041235 | 0.071794 | 0.082469 | 0.029157 | 0.176777 | 0.203063 | 0.025383 | 0.133972 | 0.025383 | 0.378929 | 0.176777 | 0.041235 |
| **Tiparp** | 0.047366 | 0.029157 | 0.017948 | 0.008974 | 0.006801 | 0.038473 | 0.002577 | 0.002244 | 0.035897 | 0.03125 | 0.002405 | 0.017948 | 0.006346 | 0.047366 | 0.003645 | 0.008373 |
| **Toe1** | 0.066986 | 0.025383 | 0.047366 | 0.038473 | 0.017948 | 0.164938 | 0.020617 | 0.008373 | 0.058315 | 0.029157 | 0.0017 | 0.076947 | 0.005921 | 0.017948 | 0.027205 | 0.017948 |
| **Trmt1** | 0.267943 | 0.094732 | 0.378929 | 0.088388 | 0.025383 | 0.164938 | 0.066986 | 0.027205 | 0.133972 | 0.5 | 0.03125 | 0.233258 | 0.054409 | 1 | 0.25 | 0.133972 |

Table S2. Continued

| **ID** | **Adrenal** | **Aorta** | **Brain** | **BAT** | **Colon** | **Heart** | **Intestine** | **Liver** | **Lung** | **Muscle** | **Skin** | **Spleen** | **Stomach** | **Testis** | **Thymus** | **WAT** |
| --- | --- | --- | --- | --- | --- | --- | --- | --- | --- | --- | --- | --- | --- | --- | --- | --- |
| **Unkl** | 0.029157 | 0.008974 | 0.038473 | 0.005524 | 0.002762 | 0.012691 | 0.006801 | 0.003173 | 0.008373 | 0.017948 | 0.002762 | 0.004487 | 0.00148 | 0.023683 | 0.014579 | 0.003906 |
| **U2af1** | 0.812252 | 0.707107 | 0.933033 | 0.659754 | 0.406126 | 0.812252 | 0.659754 | 0.153893 | 0.659754 | 1.319508 | 0.076947 | 0.5 | 0.25 | 1.071773 | 1.414214 | 0.005154 |
| **U2af1l4** | 0.287175 | 0.094732 | 0.189465 | 0.101532 | 0.03125 | 0.125 | 0.082469 | 0.007289 | 0.088388 | 0.378929 | 0.013602 | 0.094732 | 0.027205 | 0.217638 | 0.143587 | 0.082469 |
| **Zc3havl1l** | 0.003645 | 0.003645 | 0.008373 | 0.001047 | 0.002244 | 0.007289 | 0.000977 | 0.000114 | 0.007813 | 0.008373 | 0.000523 | 0.00296 | 0.001586 | 0.009618 | 0.007289 | 0.001202 |
| **Zc3h1** | 3.05E-05 | 5.69E-05 | 5.69E-05 | 1.24E-05 |  | 3.76E-05 |  | 5.03E-06 |  |  | 1.53E-05 | 5.39E-06 |  | 7.12E-06 | 9.39E-06 |  |
| **Zc3h2** | 9.25E-05 | 4.32E-05 | 3.27E-05 | 8.63E-05 | 2.31E-05 | 4.03E-05 | 4.03E-05 | 3.05E-05 | 8.05E-05 | 0.000262 | 3.51E-05 | 3.05E-05 | 1.75E-05 | 6.54E-05 | 6.1E-05 | 5.31E-05 |
| **Zc3h3** | 0.267943 | 0.082469 | 0.378929 | 0.108819 | 0.176777 | 0.707107 | 0.217638 | 0.011049 | 0.164938 | 0.035897 | 0.013602 | 0.125 | 0.047366 | 0.535887 | 0.329877 | 0.088388 |
| **Zc3h4** | 0.164938 | 0.707107 | 0.016746 | 1.741101 | 0.164938 | 1.741101 | 0.707107 | 0.143587 | 0.133972 | 0.757858 | 0.101532 | 0.133972 | 0.267943 | 0.378929 | 0.659754 | 0.217638 |
| **Zc3h5** | 0.353553 | 0.353553 | 0.466516 | 0.287175 | 0.133972 | 0.378929 | 0.189465 | 0.038473 | 0.378929 | 0.353553 | 0.020617 | 0.406126 | 0.071794 | 0.088388 | 0.353553 | 0.044194 |
| **Zc3h6** | 0.307786 | 0.066986 | 0.707107 | 0.307786 | 0.050766 | 0.287175 | 0.133972 | 0.012691 | 0.233258 | 0.615572 | 0.044194 | 0.329877 | 0.03125 | 1.071773 | 0.466516 | 0.189465 |
| **Zc3h7a** | 0.217638 | 0.287175 | 0.108819 | 0.041235 | 0.066986 | 0.011049 |  | 0.038473 | 0.406126 | 0.217638 | 0.009618 | 0.406126 | 0.116629 | 0.435275 | 0.5 | 0.03125 |
| **Zc3h7b** | 1.071773 | 0.870551 | 3.24901 | 0.659754 | 0.287175 | 1.741101 | 0.217638 | 0.125 | 0.812252 | 1.319508 | 0.022097 | 0.406126 | 0.125 | 1.866066 | 0.378929 | 0.307786 |
| **Zc3h8** | 0.044194 | 0.025383 | 0.076947 | 0.050766 | 0.011842 | 0.019237 | 0.011842 | 0.010309 | 0.025383 | 0.153893 | 0.007289 | 0.038473 | 0.006346 | 0.217638 | 0.047366 | 0.015625 |
| **Zc3h9** | 0.353553 | 0.203063 | 0.5 | 0.233258 | 0.101532 | 0.25 | 0.143587 | 0.041235 | 0.164938 | 0.233258 | 0.406126 | 0.307786 | 0.076947 | 0.329877 | 0.217638 | 0.076947 |
| **Zc3h10** | 0.019237 | 0.017948 | 0.025383 | 0.015625 | 0.007813 | 0.038473 | 0.008373 | 0.00296 | 0.013602 | 0.016746 | 0.027205 | 0.017948 | 0.003173 | 0.0625 | 0.013602 | 0.00296 |
| **Zc3h11a** | 0.0625 | 0.133972 | 0.267943 | 0.203063 | 0.017948 | 0.153893 | 0.143587 | 0.041235 | 0.153893 | 0.287175 | 0.013602 | 0.125 | 0.047366 | 0.217638 | 0.233258 | 0.133972 |
| **Zc3h12a** | 0.03125 | 0.035897 | 0.005921 | 0.153893 | 0.050766 | 0.022097 | 0.088388 | 0.004809 | 0.071794 | 0.044194 | 0.00074 | 0.25 | 0.038473 | 0.012691 | 0.133972 | 0.022097 |
| **Zc3h12b** | 0.044194 | 0.025383 | 0.25 | 0.013602 | 0.010309 | 0.025383 | 0.016746 | 0.001586 | 0.076947 | 0.0625 | 0.017948 | 0.017948 | 0.011842 | 0.094732 | 0.153893 | 0.023683 |
| **Zc3h12c** | 0.076947 | 0.015625 | 0.143587 | 0.011842 | 0.0017 | 0.076947 | 0.003906 | 0.007289 | 0.088388 | 0.054409 | 0.001953 | 0.0625 | 0.006801 | 0.133972 | 0.012691 | 0.035897 |
| **Zc3h12d** | 0.000793 | 0.003401 | 0.00015 | 0.015625 | 0.004809 | 0.000456 | 0.003173 | 0.000228 | 0.000244 | 0.001122 |  | 0.009618 | 0.000114 | 3.05E-05 | 0.005524 | 0.00037 |
| **Zc3h13** | 2.828427 | 0.233258 | 1.071773 | 0.287175 | 0.203063 | 0.189465 | 0.435275 | 0.054409 | 0.435275 | 1 | 0.003906 | 0.435275 | 0.153893 | 0.329877 | 0.659754 | 0.164938 |
| **Zc3h14** | 0.870551 | 0.870551 | 2.639016 | 1.231144 | 0.5 | 6.498019 | 0.615572 | 0.267943 | 0.707107 | 2.143547 | 0.029157 | 0.353553 | 0.076947 | 6.062866 | 0.870551 | 0.353553 |
| **Zc3h15** | 0.125 | 0.101532 | 0.615572 | 0.088388 | 0.016746 | 0.038473 | 0.038473 | 0.022097 | 0.176777 | 0.466516 | 0.015625 | 0.108819 | 0.035897 | 0.189465 | 0.066986 | 0.038473 |
| **Zfp36** | 0.466516 | 1.741101 | 0.0625 | 0.933033 | 0.757858 | 0.406126 | 1.231144 | 0.153893 | 1.866066 | 0.535887 | 0.017948 | 1.414214 | 0.287175 | 0.233258 | 0.757858 | 0.287175 |
| **Zfp36l1** | 1.148698 | 1.071773 | 0.25 | 1 | 0.233258 | 0.466516 | 0.435275 | 0.164938 | 1.148698 | 1.319508 | 0.058315 | 1.319508 | 0.133972 | 0.466516 | 0.757858 | 0.353553 |
| **Zfp36l2** | 0.466516 | 0.812252 | 0.287175 | 1.071773 | 0.353553 | 0.203063 | 0.757858 | 0.108819 | 1 | 0.933033 | 0.088388 | 0.933033 | 0.329877 | 0.267943 | 0.933033 | 0.435275 |
| **Zfp36l3** | 0.000262 | 0.000561 | 0.002405 | 0.000173 | 0.000122 | 0.000691 | 0.000523 | 4.63E-05 | 0.00074 | 0.000977 |  | 0.000488 | 9.25E-05 | 0.000793 | 0.00074 | 0.000244 |
| **Zmat5** | 0.153893 | 0.088388 | 0.153893 | 0.133972 | 0.0625 | 0.217638 | 0.076947 | 0.009618 | 0.108819 | 0.329877 | 0.004809 | 0.101532 | 0.023683 | 0.101532 | 0.125 | 0.023683 |
| **Zrsr1** | 0.005154 | 0.006801 | 0.004187 | 0.003401 | 4.03E-05 | 0.004487 | 0.000977 | 0.001289 | 0.016746 | 0.015625 | 2.31E-05 | 0.004487 | 0.001047 | 0.003906 | 0.001953 | 0.001289 |
| **Zrsr2** | 0.125 | 0.066986 | 0.101532 | 0.143587 | 0.020617 | 0.153893 | 0.143587 | 0.023683 | 0.164938 | 0.329877 | 0.009618 | 0.143587 | 0.033493 | 0.082469 | 0.133972 | 0.038473 |

Note: The values of mRNA were calculated by 2^-ΔCt^. ΔCt=Ct_gene_-Ct_cyclophilin_.
