# Supplementary material for: Genome-Wide Survey and Expression Profiling of CCCH-Zinc Finger Family Reveals a Functional Module in Macrophage Activation
Source: PLoS One. 2008 Aug 6;3(8):e2880. doi: 10.1371/journal.pone.0002880 (PMC2478707; doi:10.1371/journal.pone.0002880)
Supplement: Table S3 — (0.13 MB DOC) [file pone.0002880.s003.doc]

**Table S3. Expression Profiles of Mouse CCCH Gene Family in Macrophages**

| **ID** | **0-hour** | **0.5-hour** | **1-hour** | **2-hour** | **4-hour** | **8-hour** | **16-hour** | **24-hour** |
| --- | --- | --- | --- | --- | --- | --- | --- | --- |
| **BC003883** |  |  |  |  |  |  |  |  |
| **BC019429** |  |  |  |  |  |  |  |  |
| **Cpsf4** | 0.0883883 | 0.1166291 | 0.0625 | 0.0883883 | 0.0769465 | 0.0669858 | 0.1088188 | 0.0883883 |
| **Cpsf4l** | 0.0012886 | 0.0006011 | 0.0002804 | 0.0006011 | 0.0004556 | 0.0005609 | 0.0005609 | 0.0007401 |
| **Dhx57** | 0.0717936 | 0.0544094 | 0.0358968 | 0.0507658 | 0.0473661 | 0.0384733 | 0.0769465 | 0.0384733 |
| **Dus3l** | 0.0947323 | 0.0947323 | 0.0769465 | 0.1015315 | 0.0883883 | 0.0507658 | 0.125 | 0.0769465 |
| **Helz** | 0.2030631 | 0.1894646 | 0.1088188 | 0.1166291 | 0.1166291 | 0.0625 | 0.0507658 | 0.0441942 |
| **Leng9** | 0.0051543 | 0.0072893 | 0.0041866 | 0.0078125 | 0.0145786 | 0.0096183 | 0.015625 | 0.0126914 |
| **Mbnl1** | 2.1435469 | 1.866066 | 1.2311444 | 1.7411011 | 1.4142136 | 0.933033 | 0.6155722 | 0.5743492 |
| **Mbnl2** | 0.5743492 | 0.659754 | 0.1435873 | 0.5743492 | 0.6155722 | 0.5743492 | 0.5743492 | 0.6155722 |
| **Mbnl3** | 0.1015315 | 0.0669858 | 0.0473661 | 0.0625 | 0.0473661 | 0.0078125 | 0.0136024 | 0.0253829 |
| **Mkrn1** | 0.2871746 | 0.25 | 0.1649385 | 0.25 | 0.25 | 0.1649385 | 0.2332582 | 0.2030631 |
| **Mkrn2** | 0.0769465 | 0.0291573 | 0.0473661 | 0.0883883 | 0.0769465 | 0.03125 | 0.0824692 | 0.0717936 |
| **Mkrn3** | 0.0001726 | 0.0003966 | 0.0006443 | 0.0012023 | 0.0005609 | 0.0002278 | 0.0005609 | 0.0007401 |
| **Nhn1** | 0.1339717 | 0.1649385 | 0.0947323 | 0.1894646 | 0.1894646 | 0.1015315 | 0.1649385 | 0.1649385 |
| **Nupl2** | 0.0272047 | 0.0145786 | 0.0220971 | 0.0473661 | 0.0253829 | 0.0126914 | 0.0412346 | 0.0291573 |
| **Ppp1r10** | 0.0334929 | 0.03125 | 0.0220971 | 0.0717936 | 0.0669858 | 0.03125 | 0.0625 | 0.0625 |
| **Prr3** | 0.0206173 | 0.0167465 | 0.0096183 | 0.015625 | 0.0118415 | 0.0118415 | 0.0291573 | 0.0291573 |
| **Rbm22** | 0.1649385 | 0.2030631 | 0.1339717 | 0.1767767 | 0.2030631 | 0.0583146 | 0.1767767 | 0.1538931 |
| **Rbm26** | 0.1767767 | 0.2176376 | 0.1649385 | 0.2030631 | 0.2679434 | 0.1088188 | 0.1166291 | 0.2176376 |
| **Rbm27** | 0.0625 | 0.0507658 | 0.0358968 | 0.0507658 | 0.0583146 | 0.0412346 | 0.0384733 | 0.03125 |
| **Rc3h1** | 0.3077861 | 0.2871746 | 0.0883883 | 0.0441942 | 0.2871746 | 0.2176376 | 0.2176376 | 0.1649385 |
| **Rc3h2** | 0.2030631 | 0.1649385 | 0.0947323 | 0.1339717 | 0.2176376 | 0.2030631 | 0.1015315 | 0.1015315 |
| **Rnf113a1** | 0.0020933 | 0.0024046 | 0.0014802 | 0.0019531 | 0.0009112 | 0.0011218 | 0.0019531 | 0.0024046 |
| **Rnf113a2** | 0.0253829 | 0.0145786 | 0.0136024 | 0.0167465 | 0.0206173 | 0.0136024 | 0.0272047 | 0.0179484 |
| **Tiparp** | 0.03125 | 0.0883883 | 0.0625 | 0.2030631 | 0.3789291 | 0.1435873 | 0.0769465 | 0.1894646 |
| **Toe1** | 0.0384733 | 0.0291573 | 0.0167465 | 0.0291573 | 0.0236831 | 0.0179484 | 0.0291573 | 0.0253829 |
| **Trmt1** | 0.1767767 | 0.1649385 | 0.1166291 | 0.1767767 | 0.1435873 | 0.0769465 | 0.1538931 | 0.1649385 |
| **Unkl** | 0.0059208 | 0.0055243 | 0.0027621 | 0.0029604 | 0.0015864 | 0.0017003 | 0.0031729 | 0.0025772 |
| **U2af1** | 0.2679434 | 0.3077861 | 0.1767767 | 0.25 | 0.3077861 | 0.1894646 | 0.3077861 | 0.2679434 |
| **U2af1l4** | 0.0583146 | 0.0669858 | 0.0473661 | 0.0291573 | 0.0358968 | 0.0412346 | 0.0669858 | 0.0669858 |
| **Zc3havl1l** | 0.0027621 | 0.0031729 | 0.0014802 | 0.0015864 | 0.0012886 | 0.0003966 | 0.0006011 | 0.0006443 |
| **Zc3h1** |  |  |  |  |  |  |  |  |
| **Zc3h2** |  |  |  |  |  |  |  |  |
| **Zc3h3** | 0.0769465 | 0.0824692 | 0.0669858 | 0.0441942 | 0.1015315 | 0.0507658 | 0.1015315 | 0.125 |
| **Zc3h4** | 0.1166291 | 0.1166291 | 0.1088188 | 0.1649385 | 0.1767767 | 0.1649385 | 0.1088188 |  |
| **Zc3h5** | 0.1088188 | 0.1435873 | 0.1088188 | 0.0769465 | 0.2030631 | 0.0669858 | 0.1538931 | 0.1339717 |
| **Zc3h6** | 0.0358968 | 0.0334929 | 0.0272047 | 0.03125 | 0.0412346 | 0.0136024 | 0.0291573 | 0.0220971 |
| **Zc3h7a** | 0.0441942 | 0.0883883 | 0.0883883 | 0.1894646 | 0.329877 | 0.1166291 | 0.1166291 | 0.0625 |
| **Zc3h7b** | 0.1166291 | 0.1339717 | 0.1015315 | 0.0769465 | 0.125 | 0.0669858 | 0.1166291 | 0.0883883 |
| **Zc3h8** | 0.0179484 | 0.0118415 | 0.0089742 | 0.0089742 | 0.0078125 | 0.0068012 | 0.015625 | 0.0118415 |
| **Zc3h9** | 0.125 | 0.0824692 | 0.0769465 | 0.0583146 | 0.0669858 | 0.0441942 | 0.0824692 | 0.0717936 |
| **Zc3h10** | 0.0334929 | 0.0291573 | 0.0192366 | 0.0167465 | 0.0272047 | 0.0145786 | 0.0220971 | 0.0291573 |
| **Zc3h11a** | 0.2332582 | 0.1767767 | 0.125 | 0.1088188 | 0.1435873 | 0.1088188 | 0.1088188 | 0.1339717 |
| **Zc3h12a** | 0.1894646 | 0.3789291 | 0.6155722 | 0.5358867 | 0.8122524 | 0.659754 | 0.659754 | 0.8122524 |
| **Zc3h12b** | 0.0167465 | 0.0145786 | 0.0167465 | 0.0126914 | 0.015625 | 0.0048092 | 0.0089742 | 0.0179484 |
| **Zc3h12c** | 0.4061262 | 0.4061262 | 0.5358867 | 0.7071068 | 2.1435469 | 2.2973967 | 1.866066 | 1.6245048 |
| **Zc3h12d** | 0.0126914 | 0.0055243 | 0.0126914 | #VALUE! | 0.0027621 | 0.0063457 | 0.0014802 | 0.0012886 |
| **Zc3h13** | 0.0769465 | 0.0669858 | 0.0473661 | 0.0669858 | 0.0824692 | 0.0544094 | 0.0717936 | 0.0625 |
| **Zc3h14** | 0.5358867 | 0.3535534 | 0.2679434 | 0.3535534 | 0.25 | 0.1894646 | 0.2332582 | 0.2871746 |
| **Zc3h15** | 0.2030631 | 0.1649385 | 0.125 | 0.125 | 0.1339717 | 0.0883883 | 0.1015315 | 0.0883883 |
| **Zfp36** | 1.6245048 | 2.1435469 | 3.0314331 | 4.5947934 | 6.4980192 | 2.2973967 | 1.7411011 | 1.3195079 |
| **Zfp36l1** | 0.1894646 | 0.1339717 | 0.2176376 | 0.1339717 | 0.0824692 | 0.0291573 | 0.0179484 | 0.0236831 |
| **Zfp36l2** | 1.1486984 | 1 | 1.4142136 | 0.7578583 | 0.4352753 | 0.7071068 | 0.659754 | 1.0717735 |
| **Zfp36l3** |  |  |  |  |  |  |  |  |
| **Zmat5** | 0.3077861 | 0.2176376 | 0.2679434 | 0.1894646 | 0.4061262 | 0.2030631 | 0.2679434 | 0.3789291 |
| **Zrsr1** |  |  |  |  |  |  |  |  |
| **Zrsr2** | 0.1649385 | 0.0669858 | 0.1166291 | 0.0883883 | 0.1166291 | 0.0769465 | 0.0883883 | 0.1339717 |

Note: The values of mRNA were calculated by 2^-ΔCt^. ΔCt=Ct_gene_-Ct_cyclophilin_. Blank well means undetected.
